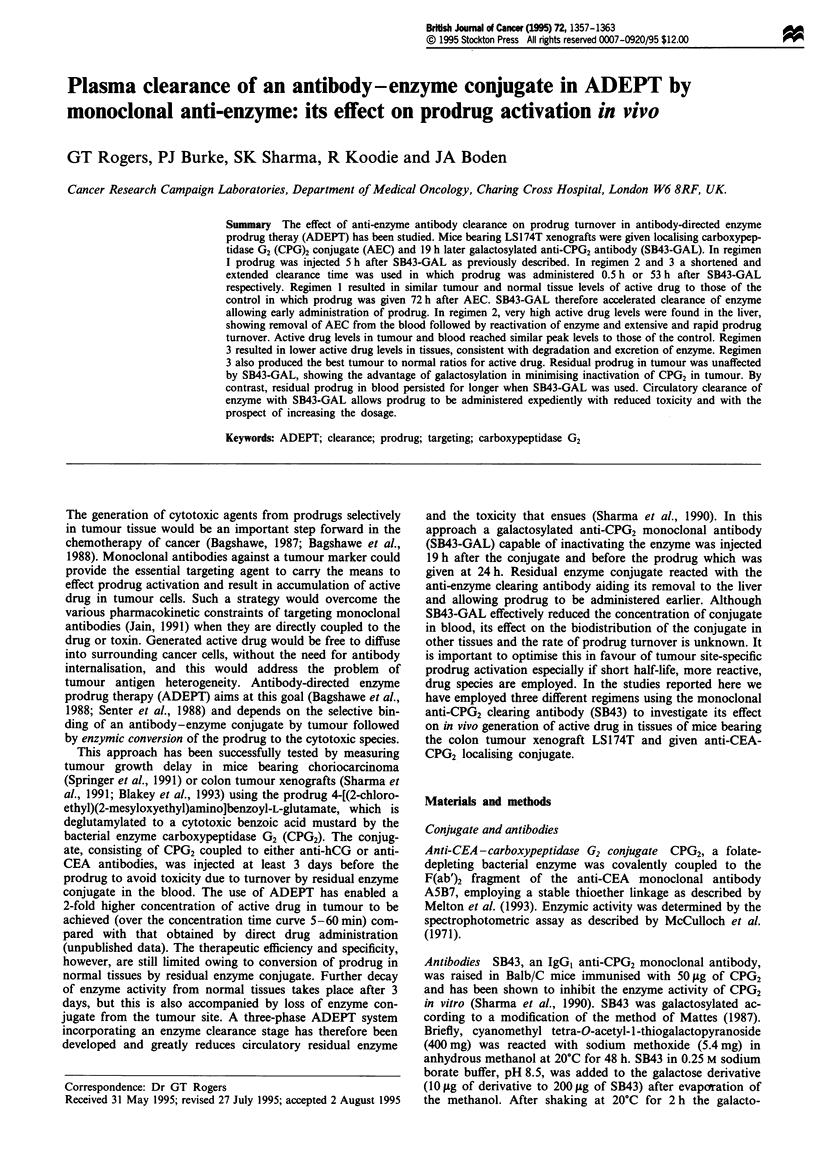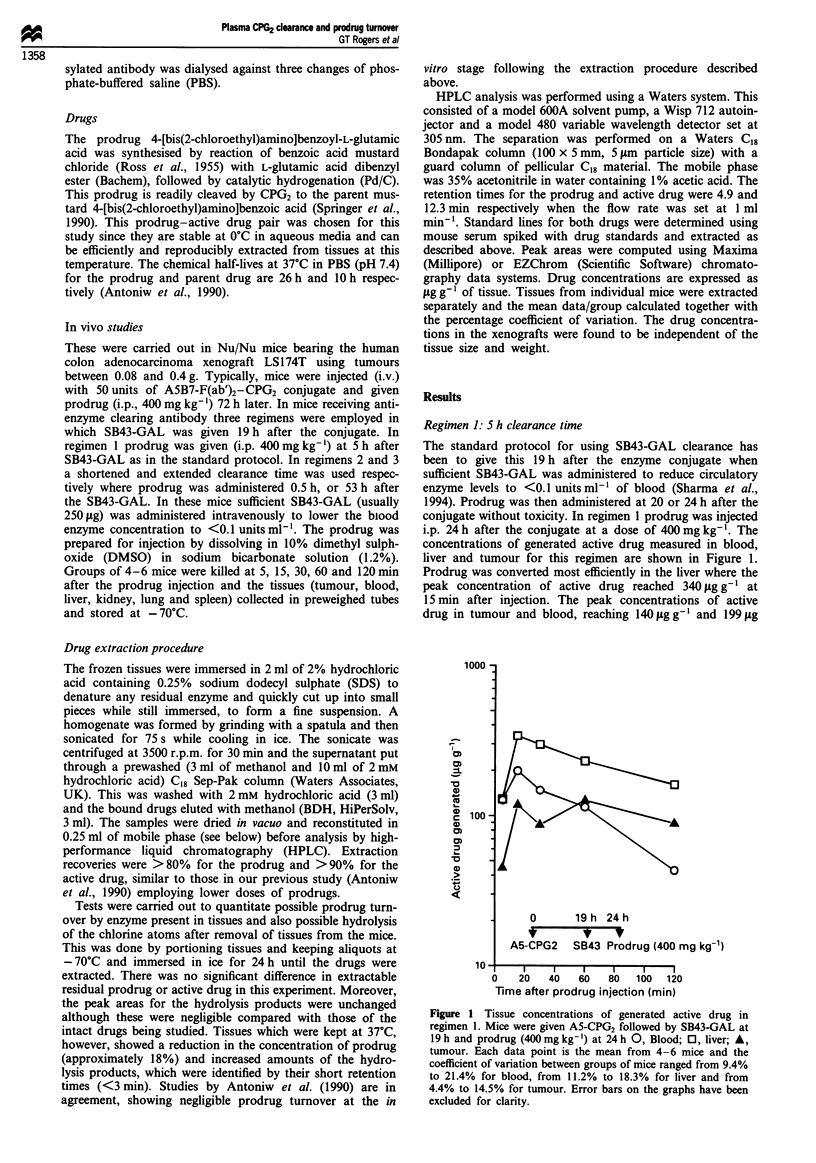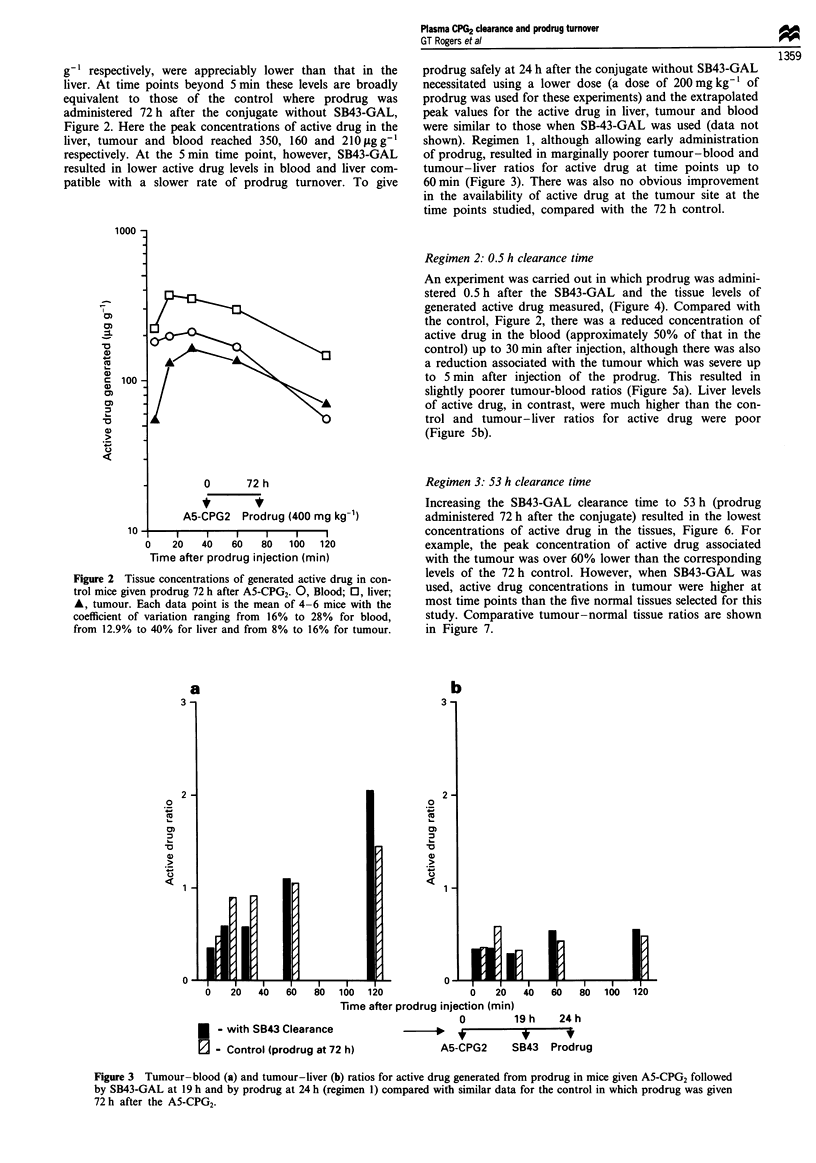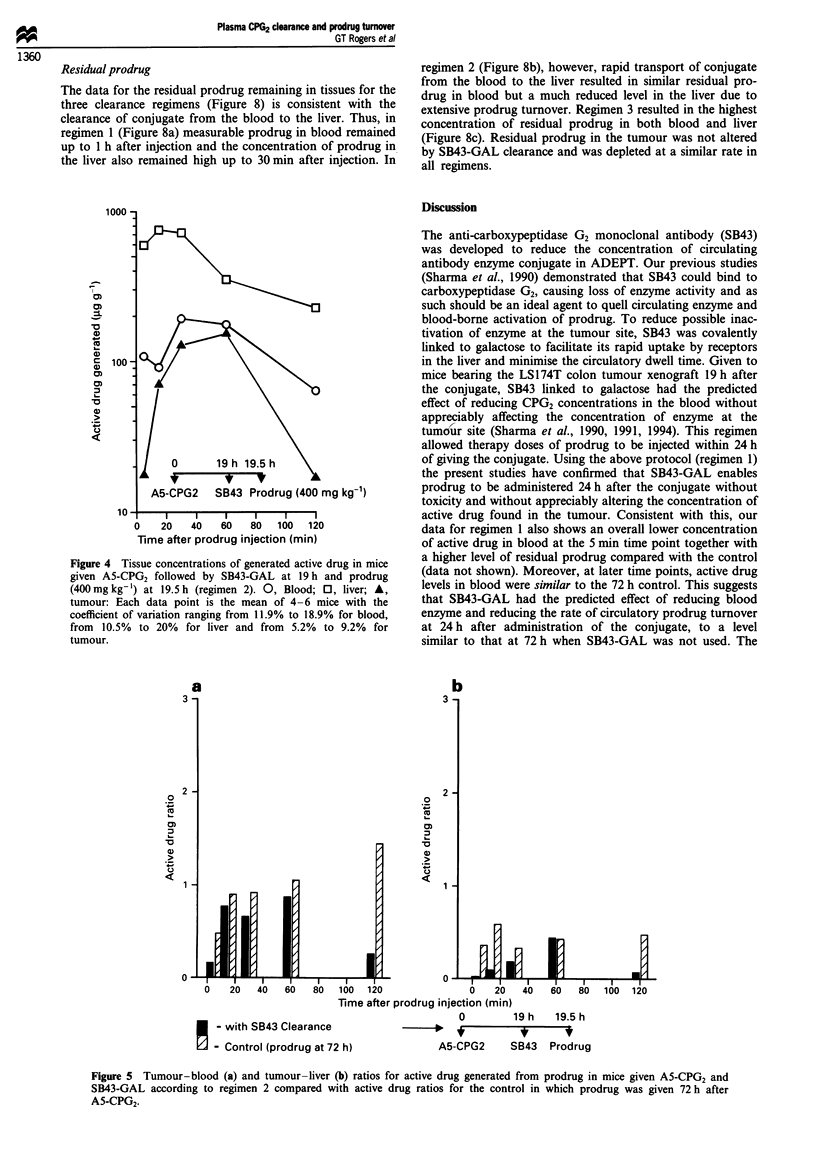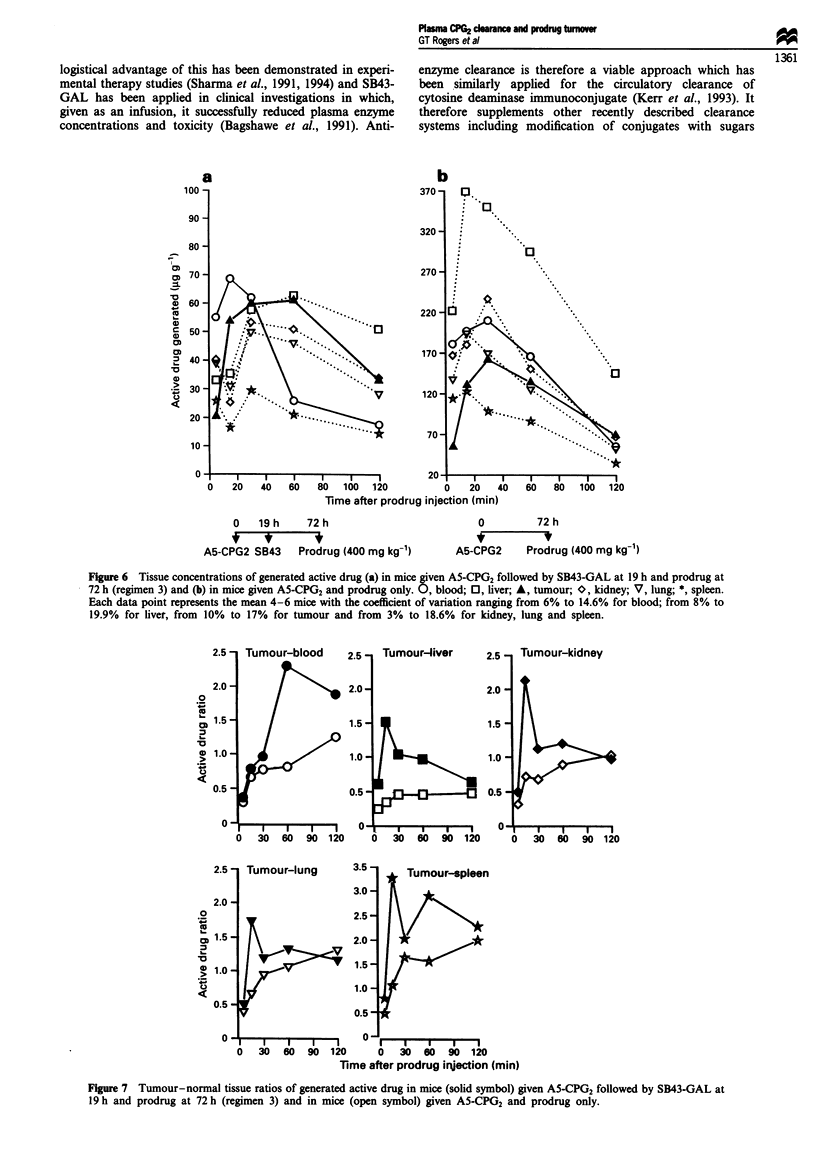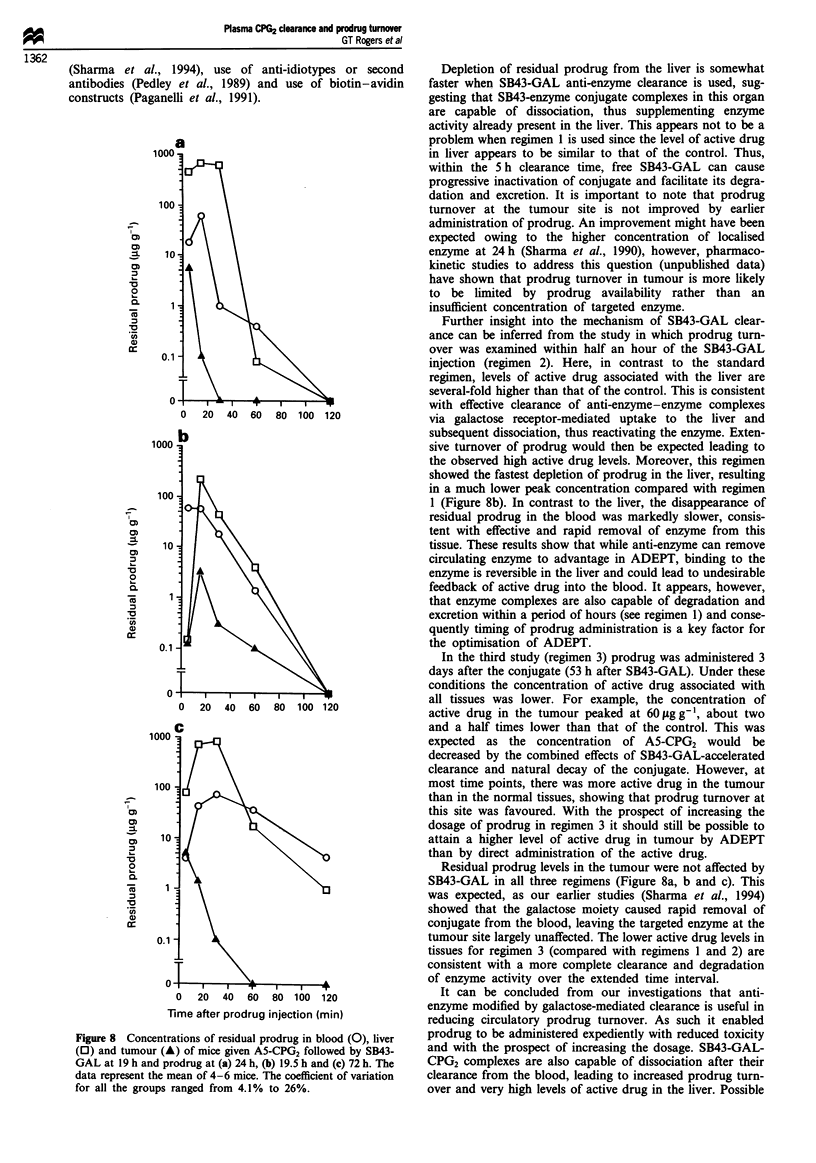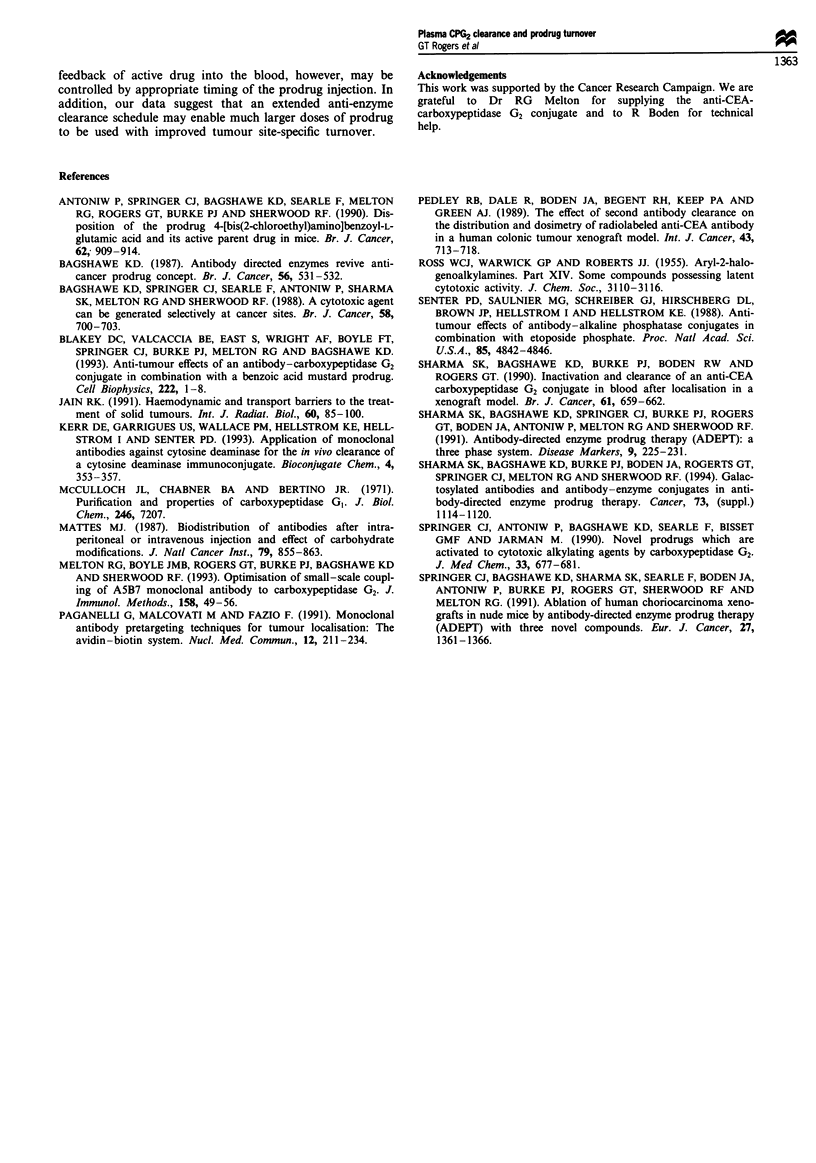# Plasma clearance of an antibody--enzyme conjugate in ADEPT by monoclonal anti-enzyme: its effect on prodrug activation in vivo.

**DOI:** 10.1038/bjc.1995.515

**Published:** 1995-12

**Authors:** G. T. Rogers, P. J. Burke, S. K. Sharma, R. Koodie, J. A. Boden

**Affiliations:** Department of Medical Oncology, Charing Cross Hospital, London, UK.

## Abstract

The effect of anti-enzyme antibody clearance on prodrug turnover in antibody-directed enzyme prodrug therapy (ADEPT) has been studied. Mice bearing LS174T xenografts were given localising carboxypeptidase G2 (CPG)2 conjugate (AEC) and 19 h later galactosylated anti-CPG2 antibody (SB43-GAL). In regimen I prodrug was injected 5 h after SB43-GAL as previously described. In regimen 2 and 3 a shortened and extended clearance time was used in which prodrug was administered 0.5 h or 53 h after SB43-GAL respectively. Regimen 1 resulted in similar tumour and normal tissue levels of active drug to those of the control in which prodrug was given 72 h after AEC. SB43-GAL therefore accelerated clearance of enzyme allowing early administration of prodrug. In regimen 2, very high active drug levels were found in the liver, showing removal of AEC from the blood followed by reactivation of enzyme and extensive and rapid prodrug turnover. Active drug levels in tumour and blood reached similar peak levels to those of the control. Regimen 3 resulted in lower active drug levels in tissues, consistent with degradation and excretion of enzyme. Regimen 3 also produced the best tumour to normal ratios for active drug. Residual prodrug in tumour was unaffected by SB43-GAL, showing the advantage of galactosylation in minimising inactivation of CPG2 in tumour. By contrast, residual prodrug in blood persisted for longer when SB43-GAL was used. Circulatory clearance of enzyme with SB43-GAL allows prodrug to be administered expediently with reduced toxicity and with the prospect of increasing the dosage.


British Journal of Cancer (1995) 72, 1357-1363
© 1995 Stockton Press All rights reserved 0007-0920/95 $12.00

### Plasma clearance of an antibody-enzyme conjugate in ADEPT by monoclonal anti-enzyme: its effect on prodrug activation *in vivo*

GT Rogers, PJ Burke, SK Sharma, R Koodie and JA Boden

*Cancer Research Campaign Laboratories, Department of Medical Oncology, Charing Cross Hospital, London W6 8RF, UK.*

**Summary** The effect of anti-enzyme antibody clearance on prodrug turnover in antibody-directed enzyme prodrug theray (ADEPT) has been studied. Mice bearing LS174T xenografts were given localising carboxypeptidase $G_2$ $(CPG)_2$ conjugate (AEC) and 19 h later galactosylated anti-$CPG_2$ antibody (SB43-GAL). In regimen I prodrug was injected 5 h after SB43-GAL as previously described. In regimen 2 and 3 a shortened and extended clearance time was used in which prodrug was administered 0.5 h or 53 h after SB43-GAL respectively. Regimen 1 resulted in similar tumour and normal tissue levels of active drug to those of the control in which prodrug was given 72 h after AEC. SB43-GAL therefore accelerated clearance of enzyme allowing early administration of prodrug. In regimen 2, very high active drug levels were found in the liver, showing removal of AEC from the blood followed by reactivation of enzyme and extensive and rapid prodrug turnover. Active drug levels in tumour and blood reached similar peak levels to those of the control. Regimen 3 resulted in lower active drug levels in tissues, consistent with degradation and excretion of enzyme. Regimen 3 also produced the best tumour to normal ratios for active drug. Residual prodrug in tumour was unaffected by SB43-GAL, showing the advantage of galactosylation in minimising inactivation of $CPG_2$ in tumour. By contrast, residual prodrug in blood persisted for longer when SB43-GAL was used. Circulatory clearance of enzyme with SB43-GAL allows prodrug to be administered expediently with reduced toxicity and with the prospect of increasing the dosage.

**Keywords:** ADEPT; clearance; prodrug; targeting; carboxypeptidase $G_2$

The generation of cytotoxic agents from prodrugs selectively in tumour tissue would be an important step forward in the chemotherapy of cancer (Bagshawe, 1987; Bagshawe *et al.*, 1988). Monoclonal antibodies against a tumour marker could provide the essential targeting agent to carry the means to effect prodrug activation and result in accumulation of active drug in tumour cells. Such a strategy would overcome the various pharmacokinetic constraints of targeting monoclonal antibodies (Jain, 1991) when they are directly coupled to the drug or toxin. Generated active drug would be free to diffuse into surrounding cancer cells, without the need for antibody internalisation, and this would address the problem of tumour antigen heterogeneity. Antibody-directed enzyme prodrug therapy (ADEPT) aims at this goal (Bagshawe *et al.*, 1988; Senter *et al.*, 1988) and depends on the selective binding of an antibody-enzyme conjugate by tumour followed by *enzymic conversion* of the prodrug to the cytotoxic species.

This approach has been successfully tested by measuring tumour growth delay in mice bearing choriocarcinoma (Springer *et al.*, 1991) or colon tumour xenografts (Sharma *et al.*, 1991; Blakey *et al.*, 1993) using the prodrug 4-[(2-chloroethyl)(2-mesyloxyethyl)amino]benzoyl-L-glutamate, which is deglutamylated to a cytotoxic benzoic acid mustard by the bacterial enzyme carboxypeptidase $G_2$ $(CPG_2)$. The conjugate, consisting of $CPG_2$ coupled to either anti-hCG or anti-CEA antibodies, was injected at least 3 days before the prodrug to avoid toxicity due to turnover by residual enzyme conjugate in the blood. The use of ADEPT has enabled a 2-fold higher concentration of active drug in tumour to be achieved (over the concentration time curve 5-60 min) compared with that obtained by direct drug administration (unpublished data). The therapeutic efficiency and specificity, however, are still limited owing to conversion of prodrug in normal tissues by residual enzyme conjugate. Further decay of enzyme activity from normal tissues takes place after 3 days, but this is also accompanied by loss of enzyme conjugate from the tumour site. A three-phase ADEPT system incorporating an enzyme clearance stage has therefore been developed and greatly reduces circulatory residual enzyme and the toxicity that ensues (Sharma *et al.*, 1990). In this approach a galactosylated anti-$CPG_2$ monoclonal antibody (SB43-GAL) capable of inactivating the enzyme was injected 19 h after the conjugate and before the prodrug which was given at 24 h. Residual enzyme conjugate reacted with the anti-enzyme clearing antibody aiding its removal to the liver and allowing prodrug to be administered earlier. Although SB43-GAL effectively reduced the concentration of conjugate in blood, its effect on the biodistribution of the conjugate in other tissues and the rate of prodrug turnover is unknown. It is important to optimise this in favour of tumour site-specific prodrug activation especially if short half-life, more reactive, drug species are employed. In the studies reported here we have employed three different regimens using the monoclonal anti-$CPG_2$ clearing antibody (SB43) to investigate its effect on *in vivo* generation of active drug in tissues of mice bearing the colon tumour xenograft LS174T and given anti-CEA-$CPG_2$ localising conjugate.

Correspondence: Dr GT Rogers
Received 31 May 1995; revised 27 July 1995; accepted 2 August 1995

### Materials and methods

### *Conjugate and antibodies*

*Anti-CEA-carboxypeptidase $G_2$ conjugate* $CPG_2$, a folate-depleting bacterial enzyme was covalently coupled to the $F(ab')_2$ fragment of the anti-CEA monoclonal antibody A5B7, employing a stable thioether linkage as described by Melton *et al.* (1993). Enzymic activity was determined by the spectrophotometric assay as described by McCulloch *et al.* (1971).

*Antibodies* SB43, an $IgG_1$ anti-$CPG_2$ monoclonal antibody, was raised in Balb/C mice immunised with 50 µg of $CPG_2$ and has been shown to inhibit the enzyme activity of $CPG_2$ *in vitro* (Sharma *et al.*, 1990). SB43 was galactosylated according to a modification of the method of Mattes (1987). Briefly, cyanomethyl tetra-*O*-acetyl-1-thiogalactopyranoside (400 mg) was reacted with sodium methoxide (5.4 mg) in anhydrous methanol at 20°C for 48 h. SB43 in 0.25 M sodium borate buffer, pH 8.5, was added to the galactose derivative (10 µg of derivative to 200 µg of SB43) after evaporation of the methanol. After shaking at 20°C for 2 h the galactosylated antibody was dialysed against three changes of phosphate-buffered saline (PBS).

### *Drugs*

The prodrug 4-[bis(2-chloroethyl)amino]benzoyl-L-glutamic acid was synthesised by reaction of benzoic acid mustard chloride (Ross *et al.*, 1955) with L-glutamic acid dibenzyl ester (Bachem), followed by catalytic hydrogenation (Pd/C). This prodrug is readily cleaved by CPG$_2$ to the parent mustard 4-[bis(2-chloroethyl)amino]benzoic acid (Springer *et al.*, 1990). This prodrug–active drug pair was chosen for this study since they are stable at 0°C in aqueous media and can be efficiently and reproducibly extracted from tissues at this temperature. The chemical half-lives at 37°C in PBS (pH 7.4) for the prodrug and parent drug are 26 h and 10 h respectively (Antoniw *et al.*, 1990).

### In vivo *studies*

These were carried out in Nu/Nu mice bearing the human colon adenocarcinoma xenograft LS174T using tumours between 0.08 and 0.4 g. Typically, mice were injected (i.v.) with 50 units of A5B7-F(ab')$_2$–CPG$_2$ conjugate and given prodrug (i.p., 400 mg kg$^{-1}$) 72 h later. In mice receiving anti-enzyme clearing antibody three regimens were employed in which SB43-GAL was given 19 h after the conjugate. In regimen 1 prodrug was given (i.p. 400 mg kg$^{-1}$) at 5 h after SB43-GAL as in the standard protocol. In regimens 2 and 3 a shortened and extended clearance time was used respectively where prodrug was administered 0.5 h, or 53 h after the SB43-GAL. In these mice sufficient SB43-GAL (usually 250 μg) was administered intravenously to lower the blood enzyme concentration to <0.1 units ml$^{-1}$. The prodrug was prepared for injection by dissolving in 10% dimethyl sulphoxide (DMSO) in sodium bicarbonate solution (1.2%). Groups of 4–6 mice were killed at 5, 15, 30, 60 and 120 min after the prodrug injection and the tissues (tumour, blood, liver, kidney, lung and spleen) collected in preweighed tubes and stored at −70°C.

### *Drug extraction procedure*

The frozen tissues were immersed in 2 ml of 2% hydrochloric acid containing 0.25% sodium dodecyl sulphate (SDS) to denature any residual enzyme and quickly cut up into small pieces while still immersed, to form a fine suspension. A homogenate was formed by grinding with a spatula and then sonicated for 75 s while cooling in ice. The sonicate was centrifuged at 3500 r.p.m. for 30 min and the supernatant put through a prewashed (3 ml of methanol and 10 ml of 2 mM hydrochloric acid) C$_{18}$ Sep-Pak column (Waters Associates, UK). This was washed with 2 mM hydrochloric acid (3 ml) and the bound drugs eluted with methanol (BDH, HiPerSolv, 3 ml). The samples were dried *in vacuo* and reconstituted in 0.25 ml of mobile phase (see below) before analysis by high-performance liquid chromatography (HPLC). Extraction recoveries were >80% for the prodrug and >90% for the active drug, similar to those in our previous study (Antoniw *et al.*, 1990) employing lower doses of prodrugs.

Tests were carried out to quantitate possible prodrug turnover by enzyme present in tissues and also possible hydrolysis of the chlorine atoms after removal of tissues from the mice. This was done by portioning tissues and keeping aliquots at −70°C and immersed in ice for 24 h until the drugs were extracted. There was no significant difference in extractable residual prodrug or active drug in this experiment. Moreover, the peak areas for the hydrolysis products were unchanged although these were negligible compared with those of the intact drugs being studied. Tissues which were kept at 37°C, however, showed a reduction in the concentration of prodrug (approximately 18%) and increased amounts of the hydrolysis products, which were identified by their short retention times (<3 min). Studies by Antoniw *et al.* (1990) are in agreement, showing negligible prodrug turnover at the *in vitro* stage following the extraction procedure described above.

HPLC analysis was performed using a Waters system. This consisted of a model 600A solvent pump, a Wisp 712 autoinjector and a model 480 variable wavelength detector set at 305 nm. The separation was performed on a Waters C$_{18}$ Bondapak column (100 × 5 mm, 5 μm particle size) with a guard column of pellicular C$_{18}$ material. The mobile phase was 35% acetonitrile in water containing 1% acetic acid. The retention times for the prodrug and active drug were 4.9 and 12.3 min respectively when the flow rate was set at 1 ml min$^{-1}$. Standard lines for both drugs were determined using mouse serum spiked with drug standards and extracted as described above. Peak areas were computed using Maxima (Millipore) or EZChrom (Scientific Software) chromatography data systems. Drug concentrations are expressed as μg g$^{-1}$ of tissue. Tissues from individual mice were extracted separately and the mean data/group calculated together with the percentage coefficient of variation. The drug concentrations in the xenografts were found to be independent of the tissue size and weight.

### Results

### *Regimen 1: 5 h clearance time*

The standard protocol for using SB43-GAL clearance has been to give this 19 h after the enzyme conjugate when sufficient SB43-GAL was administered to reduce circulatory enzyme levels to <0.1 units ml$^{-1}$ of blood (Sharma *et al.*, 1994). Prodrug was then administered at 20 or 24 h after the conjugate without toxicity. In regimen 1 prodrug was injected i.p. 24 h after the conjugate at a dose of 400 mg kg$^{-1}$. The concentrations of generated active drug measured in blood, liver and tumour for this regimen are shown in Figure 1. Prodrug was converted most efficiently in the liver where the peak concentration of active drug reached 340 μg g$^{-1}$ at 15 min after injection. The peak concentrations of active drug in tumour and blood, reaching 140 μg g$^{-1}$ and 199 μg

**Figure 1** Tissue concentrations of generated active drug in regimen 1. Mice were given A5-CPG$_2$ followed by SB43-GAL at 19 h and prodrug (400 mg kg$^{-1}$) at 24 h O, Blood; □, liver; ▲, tumour. Each data point is the mean from 4–6 mice and the coefficient of variation between groups of mice ranged from 9.4% to 21.4% for blood, from 11.2% to 18.3% for liver and from 4.4% to 14.5% for tumour. Error bars on the graphs have been excluded for clarity.

$g^{-1}$ respectively, were appreciably lower than that in the liver. At time points beyond 5 min these levels are broadly equivalent to those of the control where prodrug was administered 72 h after the conjugate without SB43-GAL, Figure 2. Here the peak concentrations of active drug in the liver, tumour and blood reached 350, 160 and 210 $\mu g\ g^{-1}$ respectively. At the 5 min time point, however, SB43-GAL resulted in lower active drug levels in blood and liver compatible with a slower rate of prodrug turnover. To give prodrug safely at 24 h after the conjugate without SB43-GAL necessitated using a lower dose (a dose of 200 mg $kg^{-1}$ of prodrug was used for these experiments) and the extrapolated peak values for the active drug in liver, tumour and blood were similar to those when SB-43-GAL was used (data not shown). Regimen 1, although allowing early administration of prodrug, resulted in marginally poorer tumour–blood and tumour–liver ratios for active drug at time points up to 60 min (Figure 3). There was also no obvious improvement in the availability of active drug at the tumour site at the time points studied, compared with the 72 h control.

**Figure 2** Tissue concentrations of generated active drug in control mice given prodrug 72 h after A5-$CPG_2$. ○, Blood; □, liver; ▲, tumour. Each data point is the mean of 4–6 mice with the coefficient of variation ranging from 16% to 28% for blood, from 12.9% to 40% for liver and from 8% to 16% for tumour.

*Regimen 2: 0.5 h clearance time*

An experiment was carried out in which prodrug was administered 0.5 h after the SB43-GAL and the tissue levels of generated active drug measured, (Figure 4). Compared with the control, Figure 2, there was a reduced concentration of active drug in the blood (approximately 50% of that in the control) up to 30 min after injection, although there was also a reduction associated with the tumour which was severe up to 5 min after injection of the prodrug. This resulted in slightly poorer tumour-blood ratios (Figure 5a). Liver levels of active drug, in contrast, were much higher than the control and tumour–liver ratios for active drug were poor (Figure 5b).

*Regimen 3: 53 h clearance time*

Increasing the SB43-GAL clearance time to 53 h (prodrug administered 72 h after the conjugate) resulted in the lowest concentrations of active drug in the tissues, Figure 6. For example, the peak concentration of active drug associated with the tumour was over 60% lower than the corresponding levels of the 72 h control. However, when SB43-GAL was used, active drug concentrations in tumour were higher at most time points than the five normal tissues selected for this study. Comparative tumour–normal tissue ratios are shown in Figure 7.

**Figure 3** Tumour–blood (a) and tumour–liver (b) ratios for active drug generated from prodrug in mice given A5-$CPG_2$ followed by SB43-GAL at 19 h and by prodrug at 24 h (regimen 1) compared with similar data for the control in which prodrug was given 72 h after the A5-$CPG_2$.

### *Residual prodrug*

The data for the residual prodrug remaining in tissues for the three clearance regimens (Figure 8) is consistent with the clearance of conjugate from the blood to the liver. Thus, in regimen 1 (Figure 8a) measurable prodrug in blood remained up to 1 h after injection and the concentration of prodrug in the liver also remained high up to 30 min after injection. In regimen 2 (Figure 8b), however, rapid transport of conjugate from the blood to the liver resulted in similar residual prodrug in blood but a much reduced level in the liver due to extensive prodrug turnover. Regimen 3 resulted in the highest concentration of residual prodrug in both blood and liver (Figure 8c). Residual prodrug in the tumour was not altered by SB43-GAL clearance and was depleted at a similar rate in all regimens.

**Figure 4** Tissue concentrations of generated active drug in mice given A5-$CPG_2$ followed by SB43-GAL at 19 h and prodrug (400 mg kg$^{-1}$) at 19.5 h (regimen 2). O, Blood; □, liver; ▲, tumour: Each data point is the mean of 4–6 mice with the coefficient of variation ranging from 11.9% to 18.9% for blood, from 10.5% to 20% for liver and from 5.2% to 9.2% for tumour.

### Discussion

The anti-carboxypeptidase $G_2$ monoclonal antibody (SB43) was developed to reduce the concentration of circulating antibody enzyme conjugate in ADEPT. Our previous studies (Sharma *et al.*, 1990) demonstrated that SB43 could bind to carboxypeptidase $G_2$, causing loss of enzyme activity and as such should be an ideal agent to quell circulating enzyme and blood-borne activation of prodrug. To reduce possible inactivation of enzyme at the tumour site, SB43 was covalently linked to galactose to facilitate its rapid uptake by receptors in the liver and minimise the circulatory dwell time. Given to mice bearing the LS174T colon tumour xenograft 19 h after the conjugate, SB43 linked to galactose had the predicted effect of reducing $CPG_2$ concentrations in the blood without appreciably affecting the concentration of enzyme at the tumour site (Sharma *et al.*, 1990, 1991, 1994). This regimen allowed therapy doses of prodrug to be injected within 24 h of giving the conjugate. Using the above protocol (regimen 1) the present studies have confirmed that SB43-GAL enables prodrug to be administered 24 h after the conjugate without toxicity and without appreciably altering the concentration of active drug found in the tumour. Consistent with this, our data for regimen 1 also shows an overall lower concentration of active drug in blood at the 5 min time point together with a higher level of residual prodrug compared with the control (data not shown). Moreover, at later time points, active drug levels in blood were *similar* to the 72 h control. This suggests that SB43-GAL had the predicted effect of reducing blood enzyme and reducing the rate of circulatory prodrug turnover at 24 h after administration of the conjugate, to a level similar to that at 72 h when SB43-GAL was not used. The

**Figure 5** Tumour–blood (**a**) and tumour–liver (**b**) ratios for active drug generated from prodrug in mice given A5-$CPG_2$ and SB43-GAL according to regimen 2 compared with active drug ratios for the control in which prodrug was given 72 h after A5-$CPG_2$.

logistical advantage of this has been demonstrated in experimental therapy studies (Sharma *et al.*, 1991, 1994) and SB43-GAL has been applied in clinical investigations in which, given as an infusion, it successfully reduced plasma enzyme concentrations and toxicity (Bagshawe *et al.*, 1991). Anti-enzyme clearance is therefore a viable approach which has been similarly applied for the circulatory clearance of cytosine deaminase immunoconjugate (Kerr *et al.*, 1993). It therefore supplements other recently described clearance systems including modification of conjugates with sugars

**Figure 6** Tissue concentrations of generated active drug (**a**) in mice given A5-$CPG_2$ followed by SB43-GAL at 19 h and prodrug at 72 h (regimen 3) and (**b**) in mice given A5-$CPG_2$ and prodrug only. O, blood; □, liver; ▲, tumour; ◇, kidney; ▽, lung; *, spleen. Each data point represents the mean 4–6 mice with the coefficient of variation ranging from 6% to 14.6% for blood; from 8% to 19.9% for liver, from 10% to 17% for tumour and from 3% to 18.6% for kidney, lung and spleen.

**Figure 7** Tumour–normal tissue ratios of generated active drug in mice (solid symbol) given A5-$CPG_2$ followed by SB43-GAL at 19 h and prodrug at 72 h (regimen 3) and in mice (open symbol) given A5-$CPG_2$ and prodrug only.

(Sharma *et al.*, 1994), use of anti-idiotypes or second antibodies (Pedley *et al.*, 1989) and use of biotin–avidin constructs (Paganelli *et al.*, 1991).

**Figure 8** Concentrations of residual prodrug in blood (○), liver (□) and tumour (▲) of mice given A5-CPG$_2$ followed by SB43-GAL at 19 h and prodrug at (**a**) 24 h, (**b**) 19.5 h and (**c**) 72 h. The data represent the mean of 4–6 mice. The coefficient of variation for all the groups ranged from 4.1% to 26%.

Depletion of residual prodrug from the liver is somewhat faster when SB43-GAL anti-enzyme clearance is used, suggesting that SB43-enzyme conjugate complexes in this organ are capable of dissociation, thus supplementing enzyme activity already present in the liver. This appears not to be a problem when regimen 1 is used since the level of active drug in liver appears to be similar to that of the control. Thus, within the 5 h clearance time, free SB43-GAL can cause progressive inactivation of conjugate and facilitate its degradation and excretion. It is important to note that prodrug turnover at the tumour site is not improved by earlier administration of prodrug. An improvement might have been expected owing to the higher concentration of localised enzyme at 24 h (Sharma *et al.*, 1990), however, pharmacokinetic studies to address this question (unpublished data) have shown that prodrug turnover in tumour is more likely to be limited by prodrug availability rather than an insufficient concentration of targeted enzyme.

Further insight into the mechanism of SB43-GAL clearance can be inferred from the study in which prodrug turnover was examined within half an hour of the SB43-GAL injection (regimen 2). Here, in contrast to the standard regimen, levels of active drug associated with the liver are several-fold higher than that of the control. This is consistent with effective clearance of anti-enzyme–enzyme complexes via galactose receptor-mediated uptake to the liver and subsequent dissociation, thus reactivating the enzyme. Extensive turnover of prodrug would then be expected leading to the observed high active drug levels. Moreover, this regimen showed the fastest depletion of prodrug in the liver, resulting in a much lower peak concentration compared with regimen 1 (Figure 8b). In contrast to the liver, the disappearance of residual prodrug in the blood was markedly slower, consistent with effective and rapid removal of enzyme from this tissue. These results show that while anti-enzyme can remove circulating enzyme to advantage in ADEPT, binding to the enzyme is reversible in the liver and could lead to undesirable feedback of active drug into the blood. It appears, however, that enzyme complexes are also capable of degradation and excretion within a period of hours (see regimen 1) and consequently timing of prodrug administration is a key factor for the optimisation of ADEPT.

In the third study (regimen 3) prodrug was administered 3 days after the conjugate (53 h after SB43-GAL). Under these conditions the concentration of active drug associated with all tissues was lower. For example, the concentration of active drug in the tumour peaked at 60 $\mu g\ g^{-1}$, about two and a half times lower than that of the control. This was expected as the concentration of A5-CPG$_2$ would be decreased by the combined effects of SB43-GAL-accelerated clearance and natural decay of the conjugate. However, at most time points, there was more active drug in the tumour than in the normal tissues, showing that prodrug turnover at this site was favoured. With the prospect of increasing the dosage of prodrug in regimen 3 it should still be possible to attain a higher level of active drug in tumour by ADEPT than by direct administration of the active drug.

Residual prodrug levels in the tumour were not affected by SB43-GAL in all three regimens (Figure 8a, b and c). This was expected, as our earlier studies (Sharma *et al.*, 1994) showed that the galactose moiety caused rapid removal of conjugate from the blood, leaving the targeted enzyme at the tumour site largely unaffected. The lower active drug levels in tissues for regimen 3 (compared with regimens 1 and 2) are consistent with a more complete clearance and degradation of enzyme activity over the extended time interval.

It can be concluded from our investigations that anti-enzyme modified by galactose-mediated clearance is useful in reducing circulatory prodrug turnover. As such it enabled prodrug to be administered expediently with reduced toxicity and with the prospect of increasing the dosage. SB43-GAL-CPG$_2$ complexes are also capable of dissociation after their clearance from the blood, leading to increased prodrug turnover and very high levels of active drug in the liver. Possible feedback of active drug into the blood, however, may be controlled by appropriate timing of the prodrug injection. In addition, our data suggest that an extended anti-enzyme clearance schedule may enable much larger doses of prodrug to be used with improved tumour site-specific turnover.

**Acknowledgements**
This work was supported by the Cancer Research Campaign. We are grateful to Dr RG Melton for supplying the anti-CEA-carboxypeptidase $G_2$ conjugate and to R Boden for technical help.

### References

ANTONIW P, SPRINGER CJ, BAGSHAWE KD, SEARLE F, MELTON RG, ROGERS GT, BURKE PJ AND SHERWOOD RF. (1990). Disposition of the prodrug 4-[bis(2-chloroethyl)amino]benzoyl-L-glutamic acid and its active parent drug in mice. *Br. J. Cancer*, **62**, 909-914.

BAGSHAWE KD. (1987). Antibody directed enzymes revive anti-cancer prodrug concept. *Br. J. Cancer*, **56**, 531-532.

BAGSHAWE KD, SPRINGER CJ, SEARLE F, ANTONIW P, SHARMA SK, MELTON RG AND SHERWOOD RF. (1988). A cytotoxic agent can be generated selectively at cancer sites. *Br. J. Cancer*, **58**, 700-703.

BLAKEY DC, VALCACCIA BE, EAST S, WRIGHT AF, BOYLE FT, SPRINGER CJ, BURKE PJ, MELTON RG AND BAGSHAWE KD. (1993). Anti-tumour effects of an antibody-carboxypeptidase $G_2$ conjugate in combination with a benzoic acid mustard prodrug. *Cell Biophysics*, **222**, 1-8.

JAIN RK. (1991). Haemodynamic and transport barriers to the treatment of solid tumours. *Int. J. Radiat. Biol.*, **60**, 85-100.

KERR DE, GARRIGUES US, WALLACE PM, HELLSTROM KE, HELLSTROM I AND SENTER PD. (1993). Application of monoclonal antibodies against cytosine deaminase for the *in vivo* clearance of a cytosine deaminase immunoconjugate. *Bioconjugate Chem.*, **4**, 353-357.

McCULLOCH JL, CHABNER BA AND BERTINO JR. (1971). Purification and properties of carboxypeptidase $G_1$. *J. Biol. Chem.*, **246**, 7207.

MATTES MJ. (1987). Biodistribution of antibodies after intraperitoneal or intravenous injection and effect of carbohydrate modifications. *J. Natl Cancer Inst.*, **79**, 855-863.

MELTON RG, BOYLE JMB, ROGERS GT, BURKE PJ, BAGSHAWE KD AND SHERWOOD RF. (1993). Optimisation of small-scale coupling of A5B7 monoclonal antibody to carboxypeptidase $G_2$. *J. Immunol. Methods.*, **158**, 49-56.

PAGANELLI G, MALCOVATI M AND FAZIO F. (1991). Monoclonal antibody pretargeting techniques for tumour localisation: The avidin-biotin system. *Nucl. Med. Commun.*, **12**, 211-234.

PEDLEY RB, DALE R, BODEN JA, BEGENT RH, KEEP PA AND GREEN AJ. (1989). The effect of second antibody clearance on the distribution and dosimetry of radiolabeled anti-CEA antibody in a human colonic tumour xenograft model. *Int. J. Cancer*, **43**, 713-718.

ROSS WCJ, WARWICK GP AND ROBERTS JJ. (1955). Aryl-2-halogenoalkylamines. Part XIV. Some compounds possessing latent cytotoxic activity. *J. Chem. Soc.*, 3110-3116.

SENTER PD, SAULNIER MG, SCHREIBER GJ, HIRSCHBERG DL, BROWN JP, HELLSTROM I AND HELLSTROM KE. (1988). Anti-tumour effects of antibody-alkaline phosphatase conjugates in combination with etoposide phosphate. *Proc. Natl Acad. Sci. U.S.A.*, **85**, 4842-4846.

SHARMA SK, BAGSHAWE KD, BURKE PJ, BODEN RW AND ROGERS GT. (1990). Inactivation and clearance of an anti-CEA carboxypeptidase $G_2$ conjugate in blood after localisation in a xenograft model. *Br. J. Cancer*, **61**, 659-662.

SHARMA SK, BAGSHAWE KD, SPRINGER CJ, BURKE PJ, ROGERS GT, BODEN JA, ANTONIW P, MELTON RG AND SHERWOOD RF. (1991). Antibody-directed enzyme prodrug therapy (ADEPT): a three phase system. *Disease Markers*, **9**, 225-231.

SHARMA SK, BAGSHAWE KD, BURKE PJ, BODEN JA, ROGERTS GT, SPRINGER CJ, MELTON RG AND SHERWOOD RF. (1994). Galactosylated antibodies and antibody-enzyme conjugates in antibody-directed enzyme prodrug therapy. *Cancer*, **73**, (suppl.) 1114-1120.

SPRINGER CJ, ANTONIW P, BAGSHAWE KD, SEARLE F, BISSET GMF AND JARMAN M. (1990). Novel prodrugs which are activated to cytotoxic alkylating agents by carboxypeptidase $G_2$. *J. Med Chem.*, **33**, 677-681.

SPRINGER CJ, BAGSHAWE KD, SHARMA SK, SEARLE F, BODEN JA, ANTONIW P, BURKE PJ, ROGERS GT, SHERWOOD RF AND MELTON RG. (1991). Ablation of human choriocarcinoma xenografts in nude mice by antibody-directed enzyme prodrug therapy (ADEPT) with three novel compounds. *Eur. J. Cancer*, **27**, 1361-1366.